# The Conserved Effector UvHrip1 Interacts with OsHGW and Infection of *Ustilaginoidea virens* Regulates Defense- and Heading Date-Related Signaling Pathway

**DOI:** 10.3390/ijms21093376

**Published:** 2020-05-10

**Authors:** Songhong Wei, Yingling Wang, Jianming Zhou, Shibo Xiang, Wenxian Sun, Xunwen Peng, Jing Li, Yingfan Hai, Yan Wang, Shuai Li

**Affiliations:** 1 College of Plant Protection, Department of Plant Pathology, Shenyang Agricultural University, Shenyang 110866, China; shw@syau.edu.cn (S.W.); 2018220411@stu.syau.edu.cn (Y.W.); 2017101428@stu.syau.edu.cn (J.Z.); 2017101251@stu.syau.edu.cn (S.X.); xunwenhn@foxmail.com (X.P.); 2018220433@stu.syau.edu.cn (J.L.); 2019220454@stu.syau.edu.cn (Y.H.); 2017500059@syau.edu.cn (Y.W.); 2 College of Plant Protection, Jilin Agricultural University, Changchun 130118, China; wxs@cau.edu.cn

**Keywords:** effector, *Ustilaginoidea virens*, flowering time, innate immunity, pathogenicity

## Abstract

*Ustilaginoidea virens*, which causes rice false smut (RFS), is one of the most detrimental rice fungal diseases and poses a severe threat to rice production and quality. Effectors in *U. virens* often act as a group of essential virulence factors that play crucial roles in the interaction between host and the pathogen. Thus, the functions of individual effectors in *U. virens* need to be further explored. Here, we demonstrated a small secreted hypersensitive response-inducing protein (hrip), named UvHrip1, which was highly conserved in *U. virens* isolates. UvHrip1 was also proven to suppress necrosis-like defense symptoms in *N. benthamiana* induced by the oomycete elicitor INF1. The localization of UvHrip1 was mainly in the nuclei and cytoplasm via monitoring the UvHrip1-GFP fusion protein in rice cells. Furthermore, Y2H and BiFC assay demonstrated that UvHrip1 interacted with OsHGW, which is a critical regulator in heading date and grain weight signaling pathways in rice. Expression patterns of defense- and heading date-related genes, *OsPR1#051* and *OsMYB21*, were down-regulated over *U. virens* infection in rice. Collectively, our data provide a theory for gaining an insight into the molecular mechanisms underlying the UvHrip1 virulence function.

## 1. Introduction

Rice false smut (RFS) caused by the ascomycetous fungus *Ustilaginoidea virens* (Cooke) Takah (teleomorph *Villosiclava virens*) is one of the most important fungal diseases in rice [1,2,3]. With heavy losses of rice production worldwide, RFS control methods have growing attention recently. *U. virens* infects the rice florets and forms false smut balls, which is covered by chlamydospore on the infected spikelets, thereby causing a significant yield loss of up to 50% around the world [4,5]. The false smut balls also contain a variety of mycotoxins, such as ustilaginoidins and ustiloxins. Twenty-six ustilaginoidins derivatives and seven ustiloxins have been isolated and identified so far. Previous reports indicated that these secondary metabolites inhibit the assembly of tubulin and mitosis of cells in eukaryotes, are toxic to humans and animals [6,7,8,9,10].

When pathogen and host plant come in contact with each other, several elicitors are released by the pathogen, as well as plant defense mechanisms are activated to combat the infection [11,12]. Pathogen-associated molecules pattern (PAMP) from the pathogen is recognized by the pathogen recognition receptor (PRR) of plant cells, and then active defense signals and trigger the PAMP-triggered immunity (PTI) [13,14]. Adapted pathogens secrete a vast array of effectors into the plant cell to hijack the plant immune system [15]. Evolutionarily, plant cells develop resistance (R) proteins, which detects and recognizes pathogen effectors specifically. Such interaction triggers rapid and robust defense responses, usually accompanied by the hypersensitive response (HR), called effector-triggered immunity (ETI) [16,17,18].

Effectors of plant pathogens were found to manipulate plant innate immunity through different molecular strategies [19]. For example, SCRE2 in *U. virens* significantly inhibits defense gene expression and oxidative burst and contributes to full virulence of *U. virens* to rice [20]. Slp1 in *Magnaporthe oryzae* and Ecp6 in *Cladosporium fulvum* competitively binds chitin with the host chitin receptors CEBiP and OsCERK, respectively, thereby drastically perturbing the host immune response triggered by chitin and promoting fungal infection [21,22]. Pit2 secreted by *Ustilago maydis* inhibits the activity of apoplastic maize cysteine proteases (CP2), the *pit2* knockout mutant is significantly attenuated in *U. maydis* virulence to host [23]. Pep1 suppresses oxidative burst driven by peroxidase POX12 and promote the infection of *U. maydis* in maize [24,25]. A lipase domain-containing protein FGL1 decreases callose formation during *Fusarium graminearum* infection through releasing free fatty acids and inhibits the activity of callose synthase, which plays an essential role in *F. graminearum* virulence [26]. Furthermore, the effectors LysM and AGLIP1, secreted by necrotrophic pathogen *Rhizoctonia solani*, inhibit chitin-induced immunity and promote pathogen infection to host [27,28]. 

Plant cell death symptoms induced by the Oomycete elicitor INF1 are physiologically similar to ETI triggered hypersensitive response. Testing the ability of inhibiting INF1-induced cell death has been a useful tool for screening the immunosuppressive ability of pathogen effectors [20,29]. In *Phytophthora sojae*, most avirulence homolog (Avh) effectors are identified to inhibit INF1-induced cell death in *Nicotiana benthamiana* [30]. INF1-triggered cell death is also suppressed by Avr3a and Pi17316, which are required for full virulence of *P. infestans* [31,32,33]. Moreover, SCREs in *U. virens* and Pst_8713 in *Puccinia striiformis* f. sp. *tritici* significantly suppresses INF1-triggered cell death in *N. benthamiana*, and play an essential role to the pathogen virulence [20,34,35].

With the help of the recently discovered genome, the molecular mechanism of *U. virens* pathogenicity was further evaluated. *U. virens* encodes at least 628 potential secreted proteins. Of these proteins, 193 are relatively small (<400 amino acids) and cysteine-rich (≥4), which have been considered as putative effectors. The cell death inhibition assays in *N. benthamiana* leaves and transcriptome analysis at different periods after pathogen infection suggest that most effectors could manipulate the plant immune responses and promote the successful colonization of pathogens in the host [2]. Furthermore, many putative effectors induce cell death or defense responses in rice and *N. benthamiana*. The signal peptides of these proteins are critical to their ability to cause cell death [36]. Collectively, many putative effectors can affect plant immunity and play a key role in *U. virens* infection. However, the functions of most effector proteins are still unknown and need to further explore.

In this study, we found a putative secreted protein named UvHrip1 (protein ID: UVI_02019870) is conserved in *U. virens* isolates. UvHrip1 suppressed INF1-triggered cell death in *N. benthamiana* leaves, and interacted with a heading date- and grain weight-related protein, OsHGW. We further showed that expression patterns of defense- and heading date-related genes, *OsPR1#051* and *OsMYB21*, were regulated during *U. virens* infection in rice. Taken together, our study suggests UvHrip1 is a functional effector and is involved in affecting host plant immunity. 

## 2. Results

### 2.1. UvHrip1 Is Highly Conserved in U. virens Isolates

**Figure 1:**
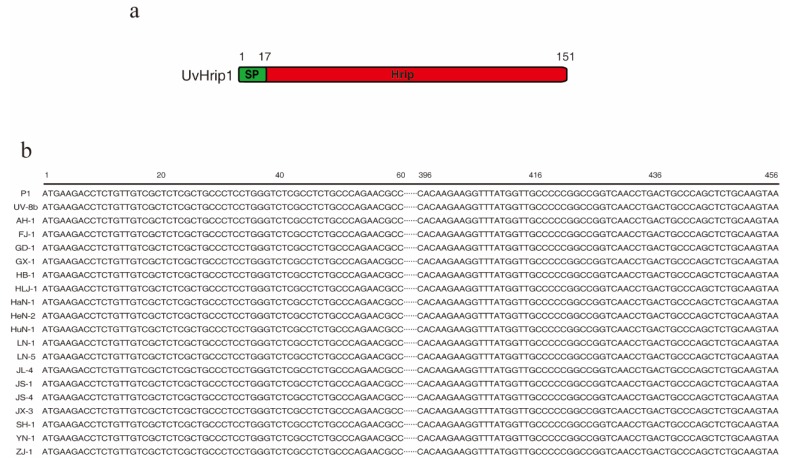
Conservation and similarity analysis of UvHrip1 from different *U. virens* isolates. (**a**) The predicted structures of UvHrip1 with 151 amino acids. SP, signal peptide; Hrip, hypersensitive response-inducing protein. (**b**) Sequence alignment of the *uvhrip1* genes from 20 different *U. virens* isolates collected from America and different regions in China. The *uvhrip1* gene sequences of UV_8b isolates were downloaded from PubMed, others were determined by sequencing.

Core effector proteins are highly conserved in many plant pathogens [28]. Based on BLAST searches by blastp against the EMBL-EBI database (https://www.ebi.ac.uk/), UvHrip1 is a hypersensitive response-inducing protein (hrip) elicitor, which has a signal peptide (SP) at the first 17 residues of N-terminal (Figure 1a). To investigate the sequence conservation of *uvhrip1*, complementary DNA (cDNA) from P1 collected in the United States and 19 isolates collected from different regions in China (Supplementary Table S1) were used as templates for polymerase chain reaction (PCR) amplification of *uvhrip1*. After sequencing, the multiple alignment analysis was performed and showed the nucleotide sequences of *uvhrip1* were all identical in the 20 isolates, indicating UvHrip1 is highly conserved in *U. virens* (Figure 1b).

### 2.2. UvHrip1 Inhibits INF1-Induced Cell Death in N. benthamiana

**Figure 2:**
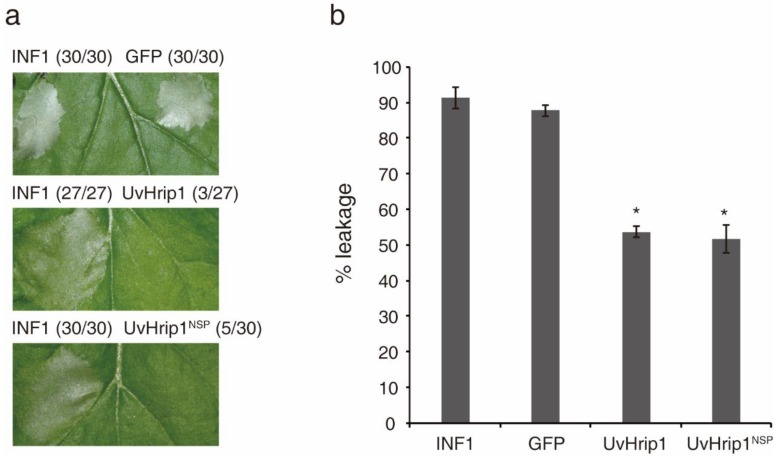
UvHrip1 suppresses INF1-triggered cell death in *N. benthamiana*. (**a**) Transiently expressed either UvHrip1 or UvHrip1^NSP^ can suppress INF1 triggered cell death in *N. benthamiana* leaves. The *Agrobacterium* strains carrying UvHrip1, UvHrip1^NSP^ and GFP were co-infiltrated with INF1 in the right sides of *N. benthamiana* leaves, respectively. The *Agrobacterium* strain carrying INF1 was infiltrated in the left sides of leaves alone. Cell death symptoms on the infiltrated leaves were photographed at 3 days. Numbers, e.g., 30/30, indicate that 30 of 30 infiltrated leaves exhibited cell death phenotypes. (**b**) Quantification of cell death in the infiltrated *N. benthamiana* leaves by measuring ion leakage. Ion leakage from the leaf discs infiltrated with different gene constructs was measured at 4 days after infiltration. The GFP constructs were infiltrated as control. Data are means ± standard error (SE) from three independent experiments. Asterisks (*) indicate *p* < 0.05.

Testing the ability of suppressing INF1-induced cell death is a useful method to identify functional effectors [37]. To investigate whether UvHrip1 regulates plant innate immunity, *Agrobacterium* strains carrying UvHrip1 and INF1 were co-infiltrated into *N. benthamiana* leaves. UvHrip1 suppresses the INF1 triggered cell death symptom in the infiltrated leaves, while green fluorescent protein (GFP) cannot. In addition, transiently expressed the UvHrip1 without SP (UvHrip1^NSP^) could also inhibit INF1 mediated cell death in *N. benthamiana* leaves (Figure 2a). Furthermore, ion leakage is correlate with cell death positively. The results showed the ion leakage of the leaves significantly reduced when co-expressing either UvHrip1 or UvHrip1^NSP^ with INF1 in comparison with that co-expressing GFP and INF1 (Figure 2b). These data demonstrated that UvHrip1 suppresses immunity-associated responses in *N. benthamiana*.

### 2.3. UvHrip1 Is Manly Localized in the Nuclei and Cytoplasm

**Figure 3:**
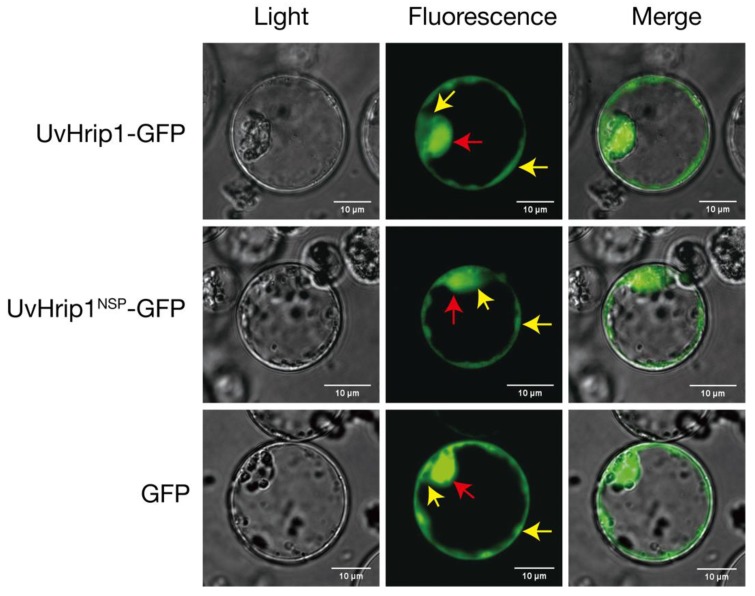
The subcellular localization of UvHrip1-GFP and UvHrip1^NSP^-GFP. UvHrip1-GFP and UvHrip1^NSP^-GFP were transiently expressed in rice protoplasts. The green fluorescence of UvHrip1-GFP and UvHrip1^NSP^-GFP was detected in the nuclei and cytoplasm of rice cells, respectively. The vector pUC19 carrying *gfp* driven by 35S promoter was used as a control. The photos were taken under a laser scanning confocal microscopy 16-20 h after transformation. The red and yellow arrows indicated nuclei and cytoplasm, respectively.

To investigate the subcellular localization of UvHrip1 *in planta*. *gfp* was fused to the C-terminal of UvHrip1 and UvHrip1^NSP^, respectively. The fusion proteins and GFP driven by the 35S promoter were transiently expressed in rice protoplasts via PEG-mediated transformation. The result showed that both green fluorescence of UvHrip1-GFP and UvHrip1^NSP^-GFP was detected in the nuclei and cytoplasm, which exhibited a similar subcellular localization of GFP transiently expressed in the rice cells (Figure 3).

### 2.4. UvHrip1 Interacts with Heading Date- and Grain Weight-Related Protein OsHGW

**Figure 4:**
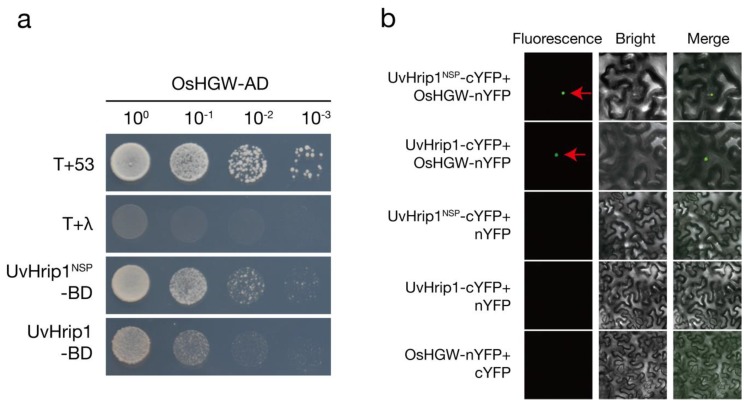
UvHrip1 interacts with OsHGW in vivo and in vitro. (**a**) Yeast two-hybrid assays revealed the interaction between UvHrip1 and OsHGW. pGADT7-*OsHGW* was co-transformed with pGBKT-*uvhrip1* or pGBKT-*uvhrip1^nsp^* in yeast strain Gold. Quadruple dropout medium (QDO) is used for interaction screening. The pGADT7-T plasmid was transformed to yeast with pGBKT7-53 or pGBKT7-Lam for positive and negative controls, respectively. T, pGADT7-T; 53, pGBKT7-53; λ, pGBKT7-Lam. (**b**) The *in planta* interaction between UvHrip1 and OsHGW was indicated by bimolecular fluorescence complementation (BiFC). Green fluorescence of *N. benthamiana* cells was observed by confocal microscopy at 3 days post agroinfiltration. The red arrows indicated nuclei.

To investigate the potential functional mechanism of UvHrip1 in rice, the yeast two-hybrid (Y2H) system was performed to preliminarily screen host proteins interacting with UvHrip1. A heading date- and grain weight-related protein, named OsHGW, was identified from a rice cDNA library when UvHrip1^NSP^ was the bait. Moreover, the interaction between full length UvHrip1 and OsHGW was further confirmed via one-to-one validation (Figure 4a). 

The in vivo interaction between UvHrip1 and OsHGW was further investigated by bimolecular fluorescence complementation (BiFC) in *N. benthamiana* leaves. OsHGW and UvHrip1 or UvHrip1^NSP^ were fused in frame with the N-terminal domain (nYFP) and C-terminal domain of yellow fluorescence protein (cYFP), respectively. When OsHGW-nYFP were co-expressed with UvHrip1-cYFP or UvHrip1^NSP^-cYFP in *N. benthamiana* leaves, the fluorescence signal was observed in the nuclei. By contrast, no fluorescence was detected in the control (Figure 4b).

Collectively, these results demonstrated that UvHrip1 interacts with OsHGW in vitro and in vivo.

### 2.5. Expression Analysis of Defense- and Heading Date-Related Genes in Young Rice Panicles during U. virens Infection 

**Figure 5:**
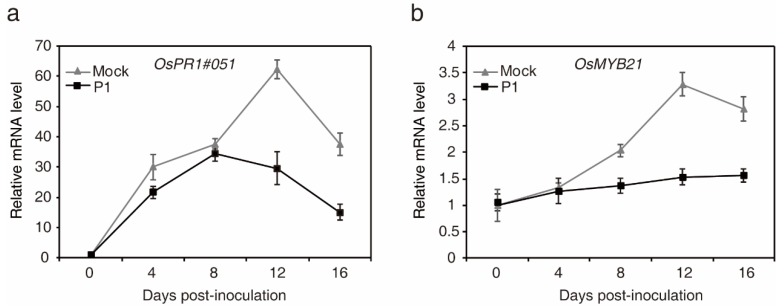
Expression analysis of defense- and heading date-related genes of rice cultivar LYP9 during *U. virens* infection. Total RNAs were prepared from rice spikelets collected at 0, 4, 8, 12 and 16 days after P1 inoculation, respectively. qRT-PCR was performed for expression profiling of *OsPR1#051* (**a**) and *OsMYB21* (**b**). The data at each time point are relative to 0 days. The expression level of *OsActin* was used as an internal reference for normalizing within the samples. Data are means ± standard error. Gene expression patterns shown are representatives from three independent repeats with similar results.

To elucidate whether the expression patterns of defense- and heading date-related genes were regulated during *U. virens* infection, the strain P1, a highly virulent isolate, was artificially inoculated into young panicles of the rice cultivar LYP9 with high P1 susceptibility [36,38]. The expression level of *OsPR1#051* and *OsMYB21* [39] were measured at 0, 4, 8, 12 and 16 days post-inoculation by quantitative real-time reverse transcription-PCR (qRT-PCR). Compared to the expression level in mock-treated plants, *OsPR1#051* and *OsMYB21* were transcriptionally inhibited during pathogen infection (Figure 5a,b). The result indicated that *U. virens* hijacks host immune response and heading date-related signaling pathway, thus facilitating infection.

## 3. Discussion

Rice false smut, caused by *U. virens*, occurs at the late stage of rice development, reduces grain yield and quality. The disease has been reported in most rice-growing areas of China and emerged as one of the major diseases in rice [1,4]. Many studies have been carried out to reduce the yield loss caused by RFS. However, little is known about the molecular mechanism underlying the interaction between rice and *U. virens*. Phytopathogenic microbes secrete the majority of effectors to regulate plant immunity by targeting different host key components [19,40]. More than 600 secreted proteins have been predicted in *U. virens* genome, 193 of which are identified as candidate effectors. Many putative effectors genes were found to be transcriptionally induced during *U. viren* infection in rice via expression profiling analyses, suggesting they may be involved in inhibiting immunity-associated responses [2]. In this study, we demonstrated that UvHrip1 is a potential effector regulating plant defense responses during pathogen infection. 

Core effector of pathogen shows a similar sequence and conserved motif across species [24,28]. BLAST searches against the EMBL-EBI database indicated UvHrip1 is a hypersensitive response-inducing protein (Hrip) elicitor, which is similar to MoHrip2 in *M. oryzae* [41], and highly conserved in *U. virens* isolates (Figure 1). Alignment analysis demonstrated that the full length of UvHrip1 and MoHrip2 showed 67% identities. The Hrip-elicitors have been identified to improve plant resistance to the pathogen, such as Hrip1 from *Alternaria tenuissima* [42], PaNie from *Pythium aphanidermatum* [43], and MoHrip1 from *M. oryzae* [41]. The defense responses are often accompanied by HR, ion influx, accumulation of nitric oxide (NO) and production of reactive oxygen species (ROS) [44]. However, no cell death symptoms were monitored within 3 days after UvHirp1-expressing *Agrobacterium* inoculated into *N. benthamiana*. Possibly, UvHirp1 induces cell death in the later time after *Agrobacterium* inoculation or perceived by specific R protein as an avirulence protein to trigger HR in the host. Therefore, the precise function of UvHirp1 will be confirmed by further experiments in rice. 

A variety of effectors secreted by biotrophic and semi-biotrophic plant pathogens can suppressed cell death in plants and are required for full virulence for infection. The ability to suppress INF1-induced cell death has been used to identify many putative functional effectors employing *Agrobacterium*-mediated transient expression assay in *N. benthamiana* [2,29,30]. In this study, we demonstrated that UvHrip1 suppresses INF1 triggered cell death in *N. benthamiana*. Moreover, the UvHrip1 truncated without signal peptide also inhibits INF1-induced cell death (Figure 2), indicating UvHrip1 may function as a cytoplasmic effector and act inside the cell. Further investigations, such as ROS assay, callose deposition, immunization-related genes expression, will be performed to confirm the plant immunity suppressing abilities of UvHrip1.

Subcellular localization detected by confocal microscopy showed that both UvHrip1-GFP and UvHrip1^NSP^-GFP were mainly localized to cytoplasm and nuclei in rice protoplasts (Figure 3). The results indicated UvHrip1 secreted by *U. virens* might have multiple functions in plant [45]. However, the multiple cellular site localization of UvHrip1 cannot be ruled out because the fusion constructs are overexpressed in rice protoplasts. Hence, the precise localization of the protein in plant cells needs to be further explored. 

The effectors have been reported to disable the plant immune system using multiple biochemical strategies and by targeting a variety of host proteins [19]. Here, the host target of UvHrip1 was screened to gain an insight into the molecular mechanisms underlying the UvHrip1 virulence function. OsHGW was initially identified to interact with UvHrip1 through the Y2H system. The in vivo interaction of UvHrip1 and OsHGW was subsequently confirmed through BiFC in *N. benthamiana* leaves (Figure 4). OsHGW contains a ubiquitin-associated (UBA) domain and regulates rice heading date and grain weight; The rice mutant *oshgw* delays heading by 20 days and reduces grain weight, but the number of grains per main panicle and the numbers of panicle per plant were not influenced; Heading date- and grain weight-related genes were differentially regulated in the *oshgw* [46]. Hence, whether UvHrip1 regulates the flower-open and immune response of rice by interacting with OsHGW remains to be further investigated.

Pathogens, which successfully colonize host tissues/organs, should have the ability to hijack or evade host immunity [47]. Here, we found the expression patterns of rice defense- and heading date-related genes, *OsPR1#051* and *OsMYB21*, were both transcriptionally inhibited over *U. virens* infection (Figure 5). *OsPR1#051* is homologous of *PR1* in *Arabidopsis*, which is associated with salicylic acid (SA) signaling pathway [39]. Rice genome encodes 12 PR1 members, all of which are transcriptionally induced during compatible and/or incompatible *M. oryzae* strains infection [48]. Our results indicated that the SA-mediated defense response and heading date-related signaling pathways in rice spikelets might play an essential role in the interaction between rice and *U. virens*. 

In summary, we identified and characterized a novel secreted protein UvHrip1 as a conserved effector that suppresses immunity in non-host plant and interacts with OsHGW, which is a key regulator in heading date and grain weight signaling pathways. However, the precise molecular mechanism of UvHrip1’s role in the interaction between rice and *U. virens* remains to be further elucidated.

## 4. Materials and methods

### 4.1. Plant Materials, Pathogen Strains and Growth Conditions

*U. virens* isolate strains were cultured using PSA medium (200 g peeled potato extract boiled in water, 20 g sucrose and 16 g agar/L). *N. benthamiana* was growth in an artificial climate chamber at 14 h light (25 ℃) / 12 h dark (23 ℃). *Agrobacterium* GV3101 and EHA105 for transient expression were cultured using LB medium (10 g tryptone, 5 g yeast extract and 10 g NaCl/L). Yeast strain Gold was cultured using YPDA medium (10 g yeast extract, 20 g peptone, 20 g glucose, 0.03 g adenine hemisulfate/L). In this study, the concentrations of antibiotics were used as follows (μg/mL): rifampin, 25; kanamycin, 50, ampicillin, 50. All data were repeated at least three times, and the results were similar. Strains and plasmids used in this study were listed in Supplementary Tables S1 and S2.

### 4.2. Plasmids Construction

The total RNA of *U. virens* was extracted using the RNA extraction kit (TaKaRa, Japan), and the concentration and quality of that were determined by NanoDrop 2000. The cDNA synthesis was performed using PrimeScript^TM^ 1st Strand cDNA Synthesis Kit (TaKaRa, Japan). The full-length and the truncated without signal peptide of UvHrip1 coding sequence amplified by *Phanta* Max ultra-fidelity DNA polymerase using the cDNA as a template. 

For INF1-induced cell death inhibition assay, *Xma* I and *Sal* I digested PCR products were subcloned into pGR107 [49]. For subcellular localization, the PCR products containing the coding sequence of UvHrip1 and UvHrip1^NSP^, was cloned into pUC19-35S-*gfp* [28] after digestion with *Bam*H I and *Sal* I, respectively. All recombination constructs were determined by sequencing. Primers used in this study are listed in Supplementary Table S3.

### 4.3. Transient Expression of Proteins in N. benthamiana Mediated by Agrobacterium

The constructed plasmid was transformed into *Agrobacterium* strains EHA105 and GV3101 by the freeze-thaw method [50]. The positive transformation was verified by PCR. The overnight cultured *Agrobacterium* carrying the correct plasmid was collected, washed 3 times with sterile double distilled water, and resuspended in 10 mM MgCl_2_ buffer (containing 10 mM MES, 10 mM acetosyringone). The optical cell density was adjusted to OD600 = 0.5 for UvHrip1- or UvHrip1^NSP^-containing strain; OD600 = 0.3 for INF1-containing strain. The *Agrobacterium* containing the corresponding plasmid was infiltrated into 4-5 weeks old *N. benthamiana* by needleless syringe. Results were observed and photographed after 3 days post-inoculation.

### 4.4. Inoculation of U. virens in Rice and qRT-PCR

Artificial inoculation was performed as described previously [20]. Briefly, P1 was cultured for 5-7 days at 120 rpm and 28 ℃ in the dark in PS medium. Mycelia and conidia were re-mixed at a concentration of 1 x 10^6^ conidia /mL with PS medium. The inocula were injected into the rice panicles by needle syringe before rice heading stage. Rice spikelets collected at 0, 4, 8, 12 and 16 days post-inoculation were stored at −80 ℃ for subsequent experiments. 

RNA extraction and cDNA synthesis were performed as described above. qRT-PCR was performed by ChamQ SYBR Color qPCR Master Mix from Vazyme Biotech Co., Ltd. and detected according to the manufacturer’s instructions by the Bio-Red CFX96 system. The internal reference gene primers used for normalizing each sample were listed in Supplementary Table S3.

### 4.5. Ion Leakage in N. benthamiana Leaf Discs

The ion leakage assay in *N. benthamiana* leaf discs to evaluate cell death was as described previously [36]. Briefly, five inoculated leaf discs of 9 mm diameter were collected and incubated in 5 mL distilled water for 3 h. The conductivity of the bathing solution was measured by a conductivity meter (FE30; Mettler Toledo). Return the leaf discs to the bathing solution, boil it in a sealed tube, and measure the conductivity of the solution again. The conductivity ratio was calculated as ion leakage.

### 4.6. Rice Protoplast Transfection and Subcellular Localization

Rice protoplasts isolation and transfection were carried out as described previously [51]. Briefly, protoplasts were transfected with the corresponding vector via polyethylene glycol (PEG)-mediated transfection after isolating from *Oryza sativa* cv. Nipponbare etiolated seedlings, and then the transfected protoplasts were incubated in solution buffer under weak light for 12-16 h.

For subcellular localization, GFP fluorescence from the overnight protoplasts was monitored using confocal microscopy (Olympus FV3000).

### 4.7. Yeast Two-Hybrid Screening

The Matchmaker^TM^ Gold yeast two-hybrid system (Clontech) was used for protein-protein interaction screening in this study [52,53]. The coding sequence of *uvhrip1^nsp^* was cloned into pGBKT7 to generate bait for screening in rice cDNA. The cDNA was synthesized by OE-Biotech Co., Ltd. (Shanghai, China). For one-to-one validation, the coding sequence of *uvhrip1* or *uvhrip1^nsp^* and *OsHGW* were cloned into pGBKT7 and pGADT7, respectively. Preparation of yeast competent cells and transformation were performed using a Frozen-EZ Yeast Transformation II Kit™ (ZYMO Research) following the manufacturer’s instructions. The constructed pGBKT7 and pGADT7 plasmids were pairwise co-transformed into the yeast strain Gold. The protein-protein interaction in yeast was analyzed on the SD double dropout (DDO, SD/-Trp-Leu) medium and SD quadruple dropout (QDO, SD/-Trp-Leu-His-Ade) medium plates. 

### 4.8. Bimolecular Fluorescence Complementation Assays

The full-length and truncated without signal peptide of *uvhrip1* were in frame fused with the 5′-end of coding sequence of YFP in pSPYNE and *OsHGW* was cloned into pSPYCE using the respective specific primers (Supplementary Table S3) [54]. The constructs were transformed into *Agrobacterium* strain EHA105 using the freeze-thaw method. Overnight-cultured *Agrobacterium* strains were collected and resuspended in induction medium (10 mM MES, pH = 5.6, 10 mM MgCl_2_ and 150 μM acetosyringone) to a final concentration of OD600 = 0.5. After incubating at room temperature for 2 h, *Agrobacterium* cultures with the pSPYNE and pSYPCE constructs were co-infiltrated into leaves of 4-5 week-old *N. benthamiana* plants. YFP or green fluorescence in the infiltrated *N. benthamiana* leaves was monitored using confocal microscopy (Olympus FV3000).

## Supplementary Materials

Supplementary materials can be found at https://www.mdpi.com/1422-0067/21/9/3376/s1.
