# Supplementary material for: The Conserved Effector UvHrip1 Interacts with OsHGW and Infection of Ustilaginoidea virens Regulates Defense- and Heading Date-Related Signaling Pathway
Source: Int J Mol Sci. 2020 May 10;21(9):3376. doi: 10.3390/ijms21093376 (PMC7246986; doi:10.3390/ijms21093376)
Supplement: Supplementary file 1 [file ijms-21-03376-s001.pdf]

Supplementary Table S1. Twenty *U. virens* isolates from USA and different provinces in China used for *uvhrip1* gene sequence analysis.

**Name**	**Rice cultivar**	**origin region**
P1	Unknown	USA
UV-8b	Zhonghua 11	Hubei
AH-1	Danjing 5	Anhui
FJ-1	Guyou 5138	Fujian
GD-1	Hemei	Guangdong
GX-1	Teyou 524	Guangxi
HB-1	Unknown	Hebei
HLJ-1	Songjing 9	Heilongjiang
HaN-1	Boyou 3050	Hainan
HeN-2	Unknown	Henan
HuN-1	Jinyou 967	Hunan
LN-1	Gangyuan	Liaoning
LN-5	Yanfeng	Liaoning
JL-4	Jijing 88	Jilin
JS-1	Huaidao 5	Jiangsu
JS-4	Wuyunjing 23	Jiangsu
JX-3	Zhongzao 27	Jiangxi
SH-1	Unknown	Shanghai
YN-1	Yunjing 39	Yunnan

ZJ-1	Zhongzheyou 86	Zhejiang

Supplementary Table S2. Strains and plasmids used in this study.

Strains/plasmids	Characteristics	References or source
Escherichia coli		
DH5a	High efficiency transformation	Lab collection
Agrobacterium		
GV3101	Wild-type, Rif$^{R, 1}$	Lab collection
EHA105	Wild-type, Rif	Lab collection
**Yeast strain**		
Gold		Clontech Co., Ltd.
**Plasmids**		
pGR107	Potato X virus（PVX）expression vector, Ka$^{R, 2}$	(Jones et al., 1999)
pGR107-*uvhrip1*		This study
pGR107- *uvhrip1*^{NSP}		This study
pGR107-*INF1*		This study
pGR107-*gfp*		This study
pUC19-*35S-gfp*	Transient expression vector for *GFP* in protoplast, Amp^{R,3}	(Li et al., 2019)
pUC19-*35S-gfp-uvhrip1*		This study
pUC19-*35S-gfp-uvhrip1*^{NSP}		This study
pGADT7	Expression vector in Yeast for Y2H assay, Amp^{R}	Clontech Co., Ltd.
pGADT7-*OsHGW*		This study
pGBKT7	Expression vector in Yeast for Y2H assay, Ka^{R}	Clontech Co., Ltd.

pGBKT7-*uvhrip1*		This study
pGBKT7-*uvhrip1*^{NSP}		This study
pSPYCE	Expression vector in *Nicotiana benthamiana* for BiFC assay, Ka^R	(Walter et al., 2004)
pSPYCE-*uvhrip1*		This study
pSPYCE-*uvhrip1*^{NSP}		This study
pSPYNE	Expression vector in *Nicotiana benthamiana* for BiFC assay, Ka^R	(Walter et al., 2004)
pSPYNE-*OsHGW*		This study

[1] Rif^R means rifampin resistance.

[2] Ka^R means kanamycin resistance.

[3] Amp^R means ampicillin resistance.

Supplementary Table S3. The designed primers used in this study.

Purpose of use	Primer name	DNA sequence
Conservative analysis	UvHrip1-F	ATGAAGACCTCTGTTGTCGC
	UvHrip1-R	GCCCAGCTCTGCAAGTAA
INF1-inducing cell death inhibiting assay	UvHrip1-NSP-pGR107-XmaI-F	TAGTGGATCCCCCGGGCAGAACGCCGTCGTCA
	UvHrip1-NSP-pGR107-SalI-R	TCATCGGCGGTCGACTTACTTGCAGAGCTGGGC
	UvHrip1-pGR107-XmaI-F	TAGTGGATCCCCCGGGCAGAACGCCGTCGTCA
	UvHrip1-pGR107-SalI-R	TCATCGGCGGTCGACTTACTTGCAGAGCTGGGC
	INF1-pGR107-XmaI-F	TAGTGGATCCCCCGGGATGAACTTTCGTGCTCTGTTCGC
	INF1-pGR107-SalI-R	TCATCGGCGGTCGACTCATAGCGACGCACACGTAGA
	GFP-pGR107-XmaI-F	TAGTGGATCCCCCGGGATGGTGAGCAAGGGCGAGG
	GFP-pGR107-SalI-R	TCATCGGCGGTCGACTTACTTGTACAGCTCGTCCATGCC
Subcellular localization	UvHrip1-NSP-pUC19-*Bam*HI-F	CGGTACCCGGGGATCCCAGAACGCCGTCGTCATCA
	UvHrip1-NSP-pUC19-SalI-R	CATGCCTGCAGGTCGACTTACTTGCAGAGCTGGGCAGT
	UvHrip1-pUC19-*Bam*HI-F	CGGTACCCGGGGATCCATGAAGACCTCTGTTGTCGCTCTC
	UvHrip1-pUC19-SalI-R	CATGCCTGCAGGTCGACTTACTTGCAGAGCTGGGCAGT
Genes expression	OsPR1#051-qPCR-F	GGGATGGGTTGGTACAAGGG
	OsPR1#051-qPCR-R	GCCAGCTTGTTATCCCACCT

	OsMYB21-qPCR-F	CCGGTGGCTGAACTATCTCC
	OsMYB21-qPCR-R	ATCTTGGACCATCGGTTGCC
	OsActin-qPCR-F	TCCATCTTGGCATCTCTCAG
	OsActin-qPCR-R	GTACCCGCATCAGGCATCTG
Y2H	OsHGW-pGADT7-F	GATTACGCTCATATGATGGACTACGACTACCGG
	OsHGW-pGADT7-R	ACCCGGGTGGAATTCCTATGATGTGCCGATCAGGTG
	UvHrip1-NSP-pGBKT7-EcoRI-F	ATGGCCATGGAGGCCGAATTCCAGAACGCCGTCGTCATCA
	UvHrip1-NSP-pGBKT7-PstI-R	CTAGTTATGCGGCCGCTGCAGGTTACTTGCAGAGCTGGGCAGT
	UvHrip1-pGBKT7-EcoRI-F	ATGGCCATGGAGGCCGAATTCATGAAGACCTCTGTTGTCGCTCTC
	UvHrip1-pGBKT7-PstI-R	CTAGTTATGCGGCCGCTGCAGGTTACTTGCAGAGCTGGGCAGT
BiFC	UvHrip1-NSP-CE-BamHI-F	GCCACTAGTGGATCCATGCAGAACGCCGTCGTCA
	UvHrip1-NSP-CE-XhoI-R	AGCGGTACCCTCGAGCTTGCAGAGCTGGGC
	UvHrip1-CE-BamHI-F	GCCACTAGTGGATCCATGAAGACCTCTGTTGTCGC
	UvHrip1-CE-XhoI-R	AGCGGTACCCTCGAGCTTGCAGAGCTGGGC
	OsHGW-NE-BamHI-F	GCCACTAGTGGATCCATGGACTACGACTACCGG
	OsHGW-NE-XhoI-R	AGCGGTACCCTCGAGTGATGTGCCGATCAGGTG
